# The role of artificial intelligence based systems for cost optimization in colorectal cancer prevention programs

**DOI:** 10.3389/frai.2022.955399

**Published:** 2022-09-30

**Authors:** Harshavardhan B. Rao, Nandakumar Bidare Sastry, Rama P. Venu, Preetiparna Pattanayak

**Affiliations:** ^1^Department of Gastroenterology, M.S. Ramaiah Medical College, Ramaiah University of Applied Sciences, Bangalore, Karnataka, India; ^2^Department of Gastroenterology, Amrita Institute of Medical Sciences and Research Centre, Kochi, Kerala, India

**Keywords:** artificial intelligence, colorectal (colon) cancer, colonoscopy, screening, cost-benefit, cost-effect analysis

## Abstract

Colorectal Cancer (CRC) has seen a dramatic increase in incidence globally. In 2019, colorectal cancer accounted for 1.15 million deaths and 24.28 million disability-adjusted life-years (DALYs) worldwide. In India, the annual incidence rates (AARs) for colon cancer was 4.4 per 100,000. There has been a steady rise in the prevalence of CRC in India which may be attributed to urbanization, mass migration of population, westernization of diet and lifestyle practices and a rise of obesity and metabolic risk factors that place the population at a higher risk of CRC. Moreoever, CRC in India differs from that described in the Western countries, with a higher proportion of young patients and more patients presenting with an advanced stage. This may be due to poor access to specialized healthcare and socio-economic factors. Early identification of adenomatous colonic polyps, which are well-recognized pre-cancerous lesions, at the time of screening colonoscopy has been shown to be the most effective measure used for CRC prevention. However, colonic polyps are frequently missed during colonoscopy and moreover, these screening programs necessitate man-power, time and resources for processing resected polyps, that may hamper penetration and efficacy in mid- to low-income countries. In the last decade, there has been significant progress made in the automatic detection of colonic polyps by multiple AI-based systems. With the advent of better AI methodology, the focus has shifted from mere detection to accurate discrimination and diagnosis of colonic polyps. These systems, once validated, could usher in a new era in Colorectal Cancer (CRC) prevention programs which would center around “Leave *in-situ*” and “Resect and discard” strategies. These new strategies hinge around the specificity and accuracy of AI based systems in correctly identifying the pathological diagnosis of the polyps, thereby providing the endoscopist with real-time information in order to make a clinical decision of either leaving the lesion *in-situ* (mucosal polyps) or resecting and discarding the polyp (hyperplastic polyps). The major advantage of employing these strategies would be in cost optimization of CRC prevention programs while ensuring good clinical outcomes. The adoption of these AI-based systems in the national cancer prevention program of India in accordance with the mandate to increase technology integration could prove to be cost-effective and enable implementation of CRC prevention programs at the population level. This level of penetration could potentially reduce the incidence of CRC and improve patient survival by enabling early diagnosis and treatment. In this review, we will highlight key advancements made in the field of AI in the identification of polyps during colonoscopy and explore the role of AI based systems in cost optimization during the universal implementation of CRC prevention programs in the context of mid-income countries like India.

## Introduction

Artificial intelligence has seamlessly integrated with Gastrointestinal endoscopy by enhancing the human capabilities combined with the infallible precision of machines. Innovations in Machine learning (ML) and computer aided detection (CADe)/computer aided diagnostic systems (CADx) have opened new paradigms that have re-defined our understanding of the world of endoscopy. Advanced imaging techniques such as Narrow Band Imaging (NBI) and pre-processing techniques like chromo-endoscopy, have provided AI-based programs a platform to create a significant impact in diagnostic endoscopy. Although AI has been widely used as a tool for better detection of pathology during the endoscopy, the shift of AI based systems to assume the role of “characterization” of the lesion in addition to locating the lesion is an exciting prospect that can have far-reaching implications in the field of endoscopy (Van Der Sommen et al., 2020). This has been mainly due to a rapid improvement in computing power, which has enabled these AI-based systems to open up novel strategies that can potentially improve cost-effectiveness and transform the endoscope into a powerful tool for preventive programs at the community level.

Colorectal cancer (CRC) is a leading cause of death with a rising incidence especially in younger age-groups, both in western countries as well as many Asian countries in the recent past (Aran et al., 2016; Deng, 2017; Mattiuzzi et al., 2019; Onyoh et al., 2019; Awedew et al., 2022; Shakuntala et al., 2022). According to GLOBOCAN 2020 data, Colorectal Cancer is the second most deadly (9.4% of total deaths) and the third most diagnosed (10.0% of total malignancies) cancer globally. Although the frequency remains higher in highly developed countries, the trend has recently stabilized or even decreased (Sung et al., 2021). However, an increase in CRC incidence and mortality has been found in medium and high human development index (HDI) countries (Deng, 2017; Veettil et al., 2017; Onyoh et al., 2019). This can be partially attributed to rapid adoption of “western” type of diets and sedentary lifestyle practices in these regions. Japan and Thailand are witnessing rapid increases in colorectal cancer incidence (Khuhaprema and Srivatanakul, 2008) and CRC incidence has been showing a steady rise in Iran over the last three decades (Dolatkhah et al., 2015). India is another country which has shown a steady rise in CRC incidence owing to changing dietary and lifestyle practices (Shakuntala et al., 2022). The treatment outcome for CRC is heavily dependent on stage at which diagnosis is established. Early-stage tumors carry a favorable prognosis with 90% survival at 5 years. However, late-stage cancers have a poor prognosis highlighting the need for screening programs that can enable early diagnosis (Färkkilä et al., 2015; Marley and Nan, 2016; Arnold et al., 2017).

CRC places a significant burden in terms of morbidity, mortality, and economic cost (Jansman et al., 2007). Previous studies conducted in high-income countries showed that the CRC imposes a high financial cost on societies and accounts for 10% of the overall economic burden of cancer. In fact, estimated economic burden to the US, England, and Korea was $14.14 billion, £542 million, and $810 million, respectively, in 2010 (Tangka et al., 2008). Essential components of the economic burden of CRC include direct medical care, nonmedical costs and productivity losses among patients and caregivers (Färkkilä et al., 2015). The healthcare expenses that are incurred by the patient and his/her family are termed out-of-pocket (OOP) expenses. Productivity costs are significant as both, the patient and his/her caregiver may have to reduce their working hours, which then results in loss of income. Moreover, self-employed patients and their caregiver(s) may occasionally have to close their business (Kolligs, 2016).

These financial considerations have been magnified by the increasing incidence of CRC in low- and middle income countries due to the growth in the aging population and rapidly changing lifestyles. Late diagnosis of CRC leads to a bad prognosis and further loss of productivity of cancer patients and caregivers thereby leading to a significant impact on the family income with downstream effects on the society as a whole (Kolligs, 2016).

In the last decade, there has been increased focus on assessing the financial burden among cancer patients and their families. For instance, subjective financial difficulty in colon cancer patients was assessed in the USA, and it was reported that 38 % of cancer patients have at least one management-related economic burden (Kolligs, 2016). One of the most cost-effective strategies of CRC management, is prevention programs using procedures like colonoscopy/sigmoidoscopy. CRC screening programs have been established in many western countries and have shown significant impact on cancer burden as well as cost-effectiveness. The objective of this paper to review the current status of Computer aided detection and diagnostic systems in CRC screening programs as viewed through the prism of financial implications on healthcare management. To that end, we will first outline the financial aspects and clinical impact of CRC screening programs in general. This will be followed by an analysis of available literature on the efficacy of CADe and CADx integration into the CRC screening programs. Finally, we will review the financial implications of these systems for CRC screening and chart a roadmap for the future of AI in CRC prevention and its potential impact on healthcare costs at the level of the individual as well as the healthcare system.

## Clinical highlights, financial aspects and a critical appraisal of CRC screening programs

Most CRC develops from pre-existing adenomas which are pre-cancerous lesions (Leslie et al., 2002). Adenomas can be detected during a colonoscopic examination of the large bowel. These adenomas are resected during the colonoscopy thereby reducing the risk of malignant transformation to CRC (Corley et al., 2014), Therefore, Adenoma Detection Rate(ADR) is an important metric for quality assessment of CRC prevention programs. Increases in ADR (by even 1%) has shown significant reduction in the rate of interval colon cancer (by around 3%) (Corley et al., 2014). Screening for colonic polyps has been instrumental in reducing CRC burden in many countries. Guidelines for screening colonoscopy with the removal of colorectal polyps every 10 years from age 50 years have been implemented in many countries in Europe and North America. Apart from training requirements for colonoscopy, optimal visualization of polyps is an area that merits further attention. Factors that can interfere with visualization of polyps during colonoscopy include those that are hidden in mucosal folds, polyps which are subtle, diminutive or transiently visible (Wang et al., 2020a). Adequate training of endoscopists to adhere to international standards of withdrawal time, quality of bowel preparation and the use of scopes that have a wider viewing angle, can be potential avenues to address these issues (Mahmud et al., 2015). However, despite this, rates of missed adenomas can be as high as 26% for polyps <5 mm in size (van Rijn et al., 2006). Adenoma Missed Rates(AMR) can be as high as 5.4% even in the case of advanced adenomas >5 mm in size (Ahn et al., 2012). In this context, AI-based systems have an important role in improving accuracy and sensitivity of colonoscopic detection and diagnosis.

### Relevant financial aspects of CRC screening programs

**Figure 1:**
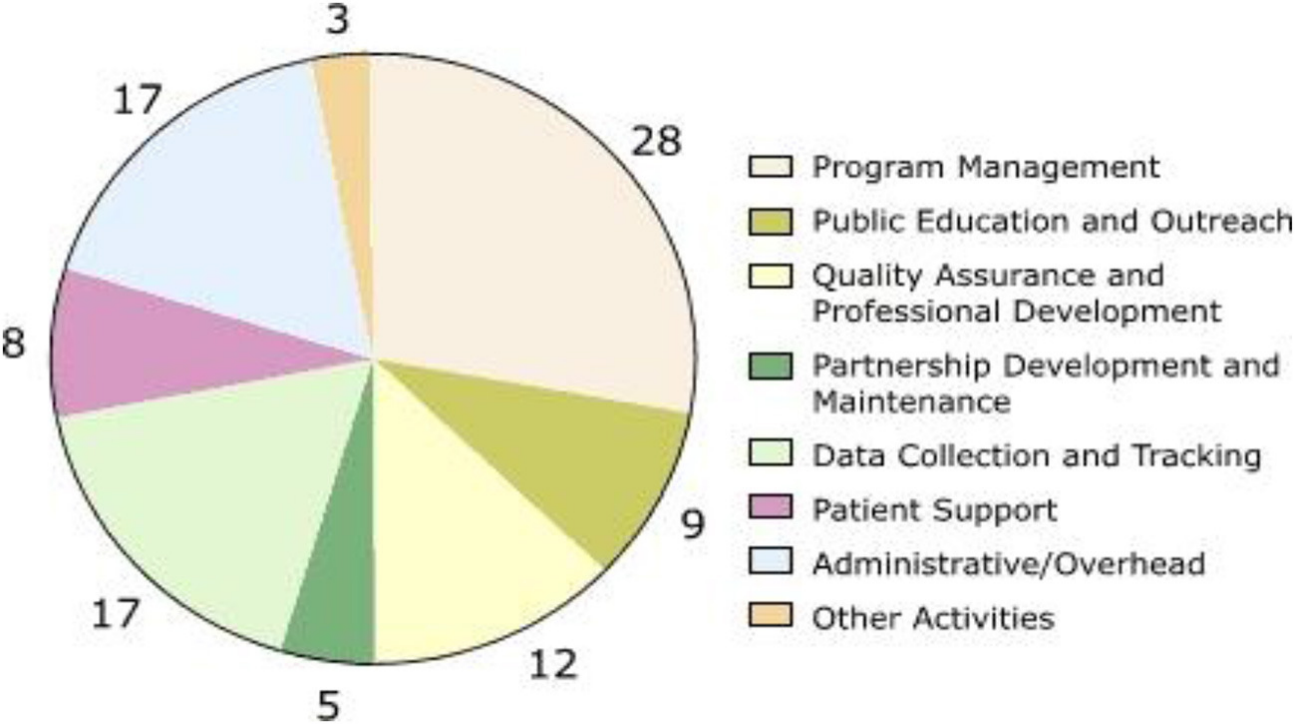
Percentage distribution of start-up costs, by activity, averaged across the five programs in the Colorectal Cancer Screening Demonstration Program, 2005–2006. Numbers do not add up to 100% due to rounding.

Screening for colorectal cancer (CRC) reduces mortality and improves the quality of life through earlier detection of precancerous polyps and thus more effective treatment of cancers. Overall costs of such programs go well-beyond the cost of the individual screening tests provided. They include expenditures to hire staff, establish contacts and partnerships with providers, develop databases and other mechanisms to maintain records and track patient outcomes, recruit patients, provide professional education, and establish medical advisory boards (Vahdatimanesh et al., 2017). Programs that provide screening services to underserved populations can incur high costs in outreach, patient education, and case management. The Centres for Disease Control and Prevention (CDC) established the Colorectal Cancer Screening Demonstration Program (CRCSDP) in 2005 to explore the feasibility of establishing a CRC screening program for the underserved U.S. population (see Figure 1).

The economic implications of colorectal cancer treatment are substantial. Factors associated with the cost of colon cancer treatment are stage of cancer, treatments that are done, places of treatment (private or public hospital), number of sessions and cycles of chemotherapy medicines used in the treatments, equipment used, and pre-colon cancer treatment (Kolligs, 2016), The treatment costs are mainly attributable to the early and terminal stages of the disease (i.e., surgery, hospitalization, chemo- and immunotherapy, and supportive care). Surgery is still the most effective treatment modality for colorectal cancer. The introduction of new chemo- and immunotherapeutics have also caused a continuing increase in treatment expenditures (Färkkilä et al., 2015; Kolligs, 2016).

The total costs to CRC include direct health care costs, informal care costs, and productivity losses. Costs of CRC are varied in various stages of the disease. Direct costs are expected to be high within 6 months of diagnosis because of operative intervention and hospitalization followed by palliative/rehabilitation care. Considering the treatment variability and intensity, India's colon cancer treatment cost varies from 1,085.82 to 9,147.52 USD. Different treatment options available for colon cancer in India are Surgery, chemotherapy, targeted therapy, and Immunotherapy, and the approximate cost is $9,147, $1,085, $4,118, and $9147, respectively (Marley and Nan, 2016) (see Table 1).

**Table 1:** Expected cost of tests and pre-colon cancer treatment in India.

**Tests**	**Description**
Health check-up	Physical examination and health check up with your doctor might cost around $8 ( 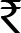 600)–$70 ( 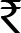 5,000)
Fecal occult blood test (FOBT)	The price range of FOBT test ranges from $5 ( 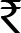 300)–$8 ( 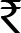 500)
Barium enema	Barium enema cost lies between $22 ( 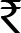 1,540)–$42 ( 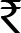 3,000)
Sigmoidoscopy	The cost of Sigmoidoscopy is from $150 ( 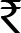 10,500)–$320 ( 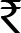 22,400)
Virtual colonoscopy	The cost of virtual colonoscopy lies between $1,400( 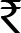 98,000)–$1750( 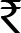 1,22,500)
Colonoscopy	The approximate cost of colonoscopy lies between $2000( 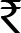 1,40,000)–$2,500( 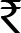 1,75,000)
Biopsy	The cost of biopsy lies between $429 ( 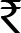 30,000)–$500 ( 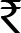 35,000).

The financial burden of cancers treatment is especially severe in developing economies like India, often forcing patients into insolvency (Mahal et al., 2013; Rahman et al., 2013). Hospital based studies done in India have shown that, on an average, a household spends USD 473.82 on cancer treatment. Since the average monthly income in the country in USD 422.18, strategies that can reduce the cancer burden, improve healthcare accessibility and manage the burgeoning costs of cancer treatment, could have a massive impact on cancer related financial burden in the country.

### Are colorectal cancer screening programs cost-effective?

In general, there is evidence that improved preventive strategies (primary and secondary) and sustainable screening practices (test or procedure used to detect disease) could reduce the cancer- related mortality by ~60% (Colditz and Wei, 2012). In the context of CRC, the most effective way of prevention has been the large-scale deployment of screening colonoscopy among average to high-risk population. Screening colonoscopy with removal of colorectal polyps reduces colorectal cancer incidence and mortality (Wolf et al., 2018). The uptake for the screening program in USA has been ~60% (Shaukat et al., 2013; Brenner et al., 2014; Zorzi et al., 2015; Lin et al., 2016; de Moor et al., 2018). Screening colonoscopy is costly and resource-intensive. However, there is evidence to show that it is cost-effective owing to the savings related to cancer treatment. In a systematic review and meta-analysis of studies that employed screening colonoscopy and sigmoidoscopy for prevention of CRC, there was a 40–60% lower risk of incident CRC and mortality (Brenner et al., 2014). In a study by Shaukat et al., patients who underwent a screening colonoscopy were followed up over a period of 30 years. Screening reduced colorectal-cancer mortality [relative risk of 0.68 with annual screening and relative risk of 0.78 with biennial screening (two yearly)] over 30 years of follow-up. This sustained reduction in risk of cancer related mortality reflects the impact of polypectomy during the screening colonoscopy procedures (Shaukat et al., 2013).

An Italian study Senore et al. (2019) published in 2019 assessed the cost-effectiveness of CRC screening programs. Using data from the Piedmont program, a Markov model was constructed to simulate the cost of screening procedures performed and weighed against the benefit of screening. The simulated screening strategies were effective in reducing incident CRC by 10–17% and were also cost-effective (Incremental cost-effectiveness ratio <1,000 euros per life year saved) (Senore et al., 2019). Even among patients who had a screen detected CRC (SD-CRC) the short term and long term outcomes were found to be much better than non-screen detected CRC (Spolverato et al., 2021). In Austria, decision-analytic cohort simulation model for colorectal adenoma and cancer with a lifelong time horizon was developed to assess the cost-effectiveness of CRC screening. Screening colonoscopy was the most effective strategy and was also found to be cost saving as compared to no screening using this model (Jahn et al., 2019).

These findings establish the pivotal role played by colonoscopy screening programs in reducing the disease burden as well as financial burden of CRC. In addition, they also establish the cost-effectiveness of colonoscopy screening in reducing cancer incidence, cancer treatment related costs and hospital admissions. However, there are inherent drawbacks to current colonoscopy protocols that can have significant downstream effects, both in terms of efficacy as well as financial ramifications.

In the subsequent sections of this article, we will review the status of artificial intelligence in CRC screening programs along with future directions for AI integrated CRC screening tools. We will review existing data on cost-effectiveness of AI-integrated solutions and propose a roadmap for optimization of CRC screening programs in the future.

## Current status of AI based systems in colonoscopy screening programs for CRC and its financial implications

The initial application of AI in endoscopy was limited to “edge detection” by identifying sharp changes in brightness, texture and “region growing” by a group of pixels of similar properties. This was useful for lesions with edges that were undetectable during standard endoscopy (Attardo et al., 2020). With the advent of advanced endoscopic imaging, subsequent Deep Neural Networks(DNN) systems could make use of additional features like texture, color, brightness and temporal factors with a high level of precision (Sánchez-Peralta et al., 2020). Subsequently, novel ML techniques were applied that could take advantage of vast datasets along with standardized image processing, to enable complex functions like accurate polyp location and classification (Yamada et al., 2019). Since, then, multiple systems have been developed that have shown improved results and accuracy (Wang et al., 2015; Misawa et al., 2016; Chen et al., 2018; Byrne et al., 2019).

Various Computer Aided Detection (CADe) systems have been applied for real-time colonoscopic detection of polyps. They have demonstrated a good accuracy for polyp detection, especially for polyps <1 cm. These systems have supplemented the endoscopist's ability to locate lesions that are obscured by debris, or poorly visualized due to specular reflections (Bernal et al., 2017). One of the first few CADe systems developed by Wang et al. was validated in a large multi-centric trial. The CADe system significantly increased mean number of adenomas per patient (0.53 vs. 0.31; *P* < 0.001) and overall ADR (29.1 vs. 20.3%; *P* < 0.001). It was also able to identify significantly more flat and sessile polyps, as well as diminutive polyps (Wang et al., 2020b). In a subsequent study by the same group, tandem colonoscopies were performed for each study participant, where the patients were randomly assigned to groups that received either routine colonoscopy or CADe assisted colonoscopy first, followed immediately by the other procedure. They found that AMR was significantly higher with routine colonoscopy (40%) than with CADe assisted colonoscopy (13.89%) (Wang et al., 2020a). Real-time CADe during screening colonoscopy, tested on several hours of colonoscopy videos, were also found to have a high accuracy of almost 97% (Urban et al., 2018). In an elegant study by Urban et al., detection of polyps was done using deep neural networks (DNN), on 8641 hand-labeled images from screening colonoscopies performed in 2,000 patients. The system was then tested on 20 random colonoscopy videos. Initially, benchmarks were developed with the help of experts who identified all polyps in the test videos. The CADe system had an accuracy of 96.5% and could detect polyps well with minimum latency (Urban et al., 2018). In a recent study by Repici et al., a novel CADe system was evaluated for real-time detection of colonic polyps. The CADe system was able to detect significantly more adenomas with an adenoma detection rate of 58% irrespective of withdrawal time. Adenomas detected per colonoscopy were also higher in the GI-Genius^TM^ group (mean 1.07 ± 1.54) than in the control group (mean 0.71 ± 1.20) (incidence rate ratio 1.46; 95% CI, 1.15–1.86). This improved ADR was mainly seen in polyps <5 mm and polyps with 5–9 mm diameter (Repici et al., 2020). These findings clearly show the utility of CADe systems in increasing the ADR and thereby reducing the rate of interval CRC.

### Impact of CADe systems on healthcare costs in screening colonoscopy

The integration of CADe systems in colonoscopic screening for polyps can have significant implications on healthcare costs associated with these programs. The additional costs of integration of AI systems with endoscopy (Table 2) needs to be off-set by the reduction of CRC related treatment costs due to reduced incidence of both CRC as well as interval cancers, detection of tumors in early stages (Carcinoma *in situ*/Stage 1) owing to higher adenoma detection rates with AI based systems. In countries like India, where out-of-pocket expenses are significant and account for a major proportion of the overall cost of treatment, measures that can address the financial aspects of a procedure that is almost universally indicated could have profound downstream implications. Unfortunately, due to the current nascent role of AI-based systems in CRC screening, there is very little data as to the objective effect of these systems on cost-effectiveness. On the one hand, considering the major healthcare expenditure is contributed by cancer treatment in most developing countries as well as emerging economies, it would follow that effective screening tools that could diagnose the cancer in a pre-malignant state will reduce cancer incidence and result in significant cost-saving (Ouakrim et al., 2015; Senore et al., 2019). As discussed in the previous section, the integration of CADe systems have shown measurable increases in ADR which would translate to significant reduction in the incidence of CRC at the population level. But from a point of view of cost-benefit analysis, however, this must off-set the increasing costs of polypectomy, histopathology evaluation of the increased number of samples that are being generated as a direct result of the CADe system.

**Table 2:** Approximate costs of integrating AI based tools into the colonoscopy screening programs.

**Detailed cost analysis for AI based tools integration with CRC screening**	**Approximate cost per procedure (USD)**
Cost of the software^*^	
A	30
B	16
**Additional cost of upgraded endoscopy processors and scopes**	
A	16
B	20
Approximate training cost	Negligible with current products on the market^**^
Total additional cost per procedure	41

* The cost of two available products in India (A & B) were obtained from manufacturers. Assuming the performance of 1,000 colonoscopies with each product, the approximate cost of AI per procedure was calculated.

** Cost of training is minimal in India since all colonoscopies are being performed by trained Gastroenterologists.

In a study published recently by Areia et al., a Markov model microsimulation was performed using colonoscopy without and with AI for CRC screening. A hypothetical cohort of 100,000 individuals aged 50–100 years; and who were at average risk for CRC(no personal or family history of colorectal cancer, adenomas, inflammatory bowel disease, or hereditary colorectal cancer syndrome) were included. Assuming a screening uptake of 60%, the initial analysis compared the hypothetical costs of screening colonoscopy with and without AI, assuming a colonoscopy was performed every 10 years, starting at age 50, until age 80 years. Individuals were followed until age 100 years. The relative reduction of incidence of CRC was found to be higher in the group employing colonoscopy with AI (48.9%) as compared to the colonoscopy without AI group (44.2%) (4.8% incremental gain). A similar trend was observed in CRC mortality which showed a 3.6% incremental gain of AI integrated colonoscopy screening. Despite the increased cost of polypectomy and histopathology evaluation, AI detection tools decreased the costs per screened individual to $3,343, from $3,400 ($57 per individual screened). In a secondary analysis, they assessed the effect of a once-in-life screening colonoscopy at age 65 years among individuals with average risk aged between 65 and 79 years. Even with this model, there were significant cost reductions observed in the microsimulation with AI-based tools as compared to conventional colonoscopy (Areia et al., 2022). This was the first study to highlight the important implications of AI detection tools on financial aspects of CRC prevention programs. It is also important to note, that the primary analysis in the study assumed a 60% uptake of screening. Assuming a 100% uptake of screening, i.e., assuming higher levels of acceptance and better penetration and accessibility of preventive programs; they found a 29.1% reduction in colorectal cancer incidence and 31.6% reduction in colorectal cancer mortality in the colonoscopy with AI scenario compared with colonoscopy without AI, resulting in a saving of $94 per person. These findings were tailored to the healthcare system and insurance costs of a single country (USA). However, similar models should be explored for country specific healthcare systems in order to demonstrate the universal effect of AI detection tools on financial aspects of CRC screening. There are inherent limitations to the microsimulation model that was adopted to demonstrate the cost saving aspect of AI detection tools. These limitations include many assumptions on the patient behavior, acceptance, and implementation of screening programs. However, these limitations notwithstanding, it highlights a very intriguing area that can inform future efforts to integrate AI detection tools in our everyday practice.

### The role of CADx system as an additional cost-saving strategy

As opposed to CADe systems, CADx systems can characterize the polyps as neoplastic or hyperplastic based on the AI diagnostic tool adopted (Rodriguez-Diaz et al., 2021). These systems are still under intense study and the routine implementation of which, could be a potentially disruptive technology that could usher in a new age in CRC screening programs. Essentially, the advanced diagnostic capabilities of CADx systems could open up the possibilities for two alternate strategies in CRC screening—“Resect and discard” and “Leave *in situ*” (Rex et al., 2011; Ladabaum et al., 2013). An adenomatous, diminutive polyp diagnosed by a CADx system which has a good accuracy, could just be resected and discarded, obviating the need for histopathology evaluation. In addition, a hyperplastic/mucosal polyp as diagnosed by CADx system in real time (i.e., during the colonoscopy) could potentially be left *in situ* as they have no malignant potential. This will drastically reduce the costs associated with polypectomy and histopathology evaluation of the biopsy samples that are otherwise the standard of care. Additionally, these measures could have far-reaching implications in logistical considerations of CRC screening programs, reduction of man-power and specialized equipment thereby increasing the operational efficiency, penetration, accessibility, and uptake of these programs. As demonstrated in the study by Areia et al. (2022), the uptake of screening could have profound effects on the ADR rate and subsequently on the cost-effectiveness of the CRC screening program.

The clinical application of optical diagnosis especially for diminutive polyps is increasingly being considered as the “next step” in colonoscopic screening of polyps (Ignjatovic et al., 2009; Hassan et al., 2010; Kessler et al., 2011). The National Institute for Clinical and Healthcare Excellence (NICE), which is responsible for setting the clinical standards in the United Kingdom (UK), approved the optical diagnosis of diminutive colorectal polyps using narrow-spectrum endoscopy in 2017. This was a significant step forward toward its implementation in clinical practice (National Institute for Health Clinical Excellence, 2017). However, this has not been widely used owing to its lack of specificity in non-expert hands. That is why, a reliable CADx system with good accuracy, fidelity and low latency could be the ideal alternative for optimal diagnosis of polyps.

Initially, CADx systems were able to differentiate between adenomatous from hyperplastic polyps while employing advanced image processing techniques like magnification chromoendoscopy or magnification NBI (Tischendorf et al., 2010; Gross et al., 2011; Kominami et al., 2016). However, these studies used AI techniques that were sub-optimal which limited its real-time application owing to the requirement complex post procedure image processing like manual segmentation of polyp margins and magnification techniques that are not widely available. The advent of DNN techniques changed the scenario and the newer CADx systems could diagnose polyps with minimal latency. In a prospective study of 41 patients, a CADx system was testes for diagnosing adenomatous polyps. The system showed a diagnostic accuracy of 93.2% for a real-time diagnosis on 118 colorectal lesions which was evaluated with NBI. Among the patients with small polyps, an impressive 92.7% showed concordance between the CADx diagnosis and the pathological findings (Zachariah et al., 2020). In another intriguing study, a novel CADx system was able to improve the overall accuracy of polyp diagnosis to 88.5% from 82.5% among controls (*P* < 0.05). This effect was especially pronounced among novices with limited training in using enhanced imaging techniques for polyp characterization, where the accuracy jumped from 73.8 to 85.6% (Jin et al., 2020). These findings indicate the feasibility of implementation of CADx systems in clinical practice.

A recent multicentric study, published by Mori and colleagues attempts to explore the financial implications of a novel CADx system (Mori et al., 2020). In this study, an add-on analysis was performed on a clinical trial that assessed the efficacy of a novel CADx system in differentiating neoplastic polyps from non-neoplastic polyps. The average cost was estimated for two situations, namely a Leave *in situ* strategy for supported by the AI prediction for diminutive rectosigmoid polyps, and a resect-all-polyps strategy. The gross annual costs for screening colonoscopies were considered based on data provided under public health insurances in 4 different countries. The novel CADx system could correctly differentiate neoplastic polyps with 93.3% sensitivity, 95.2% specificity, and 95.2% negative predictive value. This resulted in 105 polyps which were removed and 145 polyps which were left *in situ*. These strategies led to significant reductions of the average colonoscopy cost and the gross annual reimbursement for colonoscopies by 6.9% and 12.3 million dollars in England, 18.9% and 149.2 million dollars in Japan, 10.9% and 85.2 million dollars in the United States, and 7.6% and 1.1 million dollars in Norway compared to the resect-all-polyps strategy. This study clearly demonstrates the impact CADx systems could have on healthcare costs for CRC prevention programs and merits further studies to establish its role as an indispensable tool in CRC prevention programs worldwide.

## Conclusions and future directions

AI-based detection tools and CADx systems are the way forward in CRC prevention. These tools can not only reduce the incidence of CRC through improved ADR, but it can have profound implications on cost reduction. This would result in better results in cost-effectiveness analysis and have far-reaching implications in mid-income and emerging economies like India. Apart from establishing the validity of optical diagnosis of polyps using CADx systems, future studies that utilize AI tools to predict the surveillance interval for colonoscopy for individual polyps based on morphological and clinical characteristics could represent a paradigm shift in our standard practices for CRC screening. This could help in re-allocation of resources in an efficient and streamlined manner so as to ensure the right patients get screened regularly, while patients at low risk of recurrence need not be subjected to repeated procedures (Mori et al., 2018, 2020). Cost reduction by using this strategy, if established could be an additional benefit while still maintaining efficacy and ADR at levels higher than what is currently been observed in most countries.

Additional studies that attempt to collate the total cost savings from implementation of CADx systems at the community level by obviating the need for histopathological correlation while adopting strategies like “Resect and discard” and “Leave *in situ*” needs to be performed either by simulation modeling or by longitudinal studies could highlight the true impact of AI based tools on CRC screening. In India, these tools could have incremental effects in addition to cost reduction by reducing the resources (human and equipment), infrastructure and logistical roadblocks that currently hamper the accessibility of CRC screening programs across demographics. Since CRC has shown a relentless upward trend in India, the time to consider organized screening programs aligning with the National Digital Health Mission is essential. The integration of AI tools for detection and characterization of colonic polyps with its significant cost reduction and additional benefits with regard to healthcare financial management, could be exactly what is needed to push for a national CRC prevention program (Table 3).

**Table 3:** Relevant classification of important references cited in the article.

**Categories of references**	**References**
CRC epidemiology, burden and prevalence	Khuhaprema and Srivatanakul, 2008; Corley et al., 2014; Dolatkhah et al., 2015; Aran et al., 2016; Kolligs, 2016; Marley and Nan, 2016; Arnold et al., 2017; Deng, 2017; Veettil et al., 2017; Mattiuzzi et al., 2019; Onyoh et al., 2019; Sung et al., 2021; Awedew et al., 2022; Shakuntala et al., 2022
Cost benefit analysis of CRC screening	Jansman et al., 2007; Tangka et al., 2008; Mahal et al., 2013; Rahman et al., 2013; Brenner et al., 2014; Färkkilä et al., 2015; Zorzi et al., 2015; Vahdatimanesh et al., 2017; de Moor et al., 2018; Jahn et al., 2019; Senore et al., 2019
AI based tools for detection of polyps during screening colonoscopy	Wang et al., 2015, 2020a,b; Bernal et al., 2017; Urban et al., 2018; Attardo et al., 2020; Repici et al., 2020; Sánchez-Peralta et al., 2020
AI based tools for Diagnosis of polyps during screening colonoscopy	Ignjatovic et al., 2009; Tischendorf et al., 2010; Gross et al., 2011; Ladabaum et al., 2013; Kominami et al., 2016; Misawa et al., 2016; National Institute for Health Clinical Excellence, 2017; Chen et al., 2018; Mori et al., 2018; Byrne et al., 2019; Yamada et al., 2019; Jin et al., 2020; Zachariah et al., 2020; Rodriguez-Diaz et al., 2021
Cost benefit analysis of AI based systems	Hassan et al., 2010; Kessler et al., 2011; Mori et al., 2020; Areia et al., 2022

## Author contributions

HR and PP performed the literature review and drafted the manuscript. NS reviewed the manuscript and provided important epidemiological insights. RV reviewed the final manuscript for critical insights. All authors contributed to the article and approved the submitted version.

## Conflict of interest

The authors declare that the research was conducted in the absence of any commercial or financial relationships that could be construed as a potential conflict of interest.

## Publisher's note

All claims expressed in this article are solely those of the authors and do not necessarily represent those of their affiliated organizations, or those of the publisher, the editors and the reviewers. Any product that may be evaluated in this article, or claim that may be made by its manufacturer, is not guaranteed or endorsed by the publisher.
